# Impacts of dialysis adequacy and intradialytic hypotension on changes in dialysis recovery time

**DOI:** 10.1186/s12882-020-02187-9

**Published:** 2020-12-07

**Authors:** Murilo Guedes, Roberto Pecoits-Filho, Juliana El Ghoz Leme, Yue Jiao, Jochen G. Raimann, Yuedong Wang, Peter Kotanko, Thyago Proença de Moraes, Ravi Thadhani, Franklin W. Maddux, Len A. Usvyat, John W. Larkin

**Affiliations:** 1 School of Medicine, Pontifícia Universidade Católica do Paraná, Curitiba, PR Brazil; 2 Global Medical Office, Fresenius Medical Care, 920 Winter Street, Waltham, MA 02451 USA; 3 Research Division, Renal Research Institute, New York, NY USA; 4 University of California Santa Barbara, Santa Barbara, CA USA; 5 Icahn School of Medicine at Mount Sinai, New York, NY USA; 6 Partners HealthCare, Boston, MA USA

**Keywords:** Dialysis recovery time, Patient reported outcomes, Health related quality of life, Adequacy, Intradialytic hypotension

## Abstract

**Background:**

Dialysis recovery time (DRT) surveys capture the perceived time after HD to return to performing regular activities. Prior studies suggest the majority of HD patients report a DRT > 2 h. However, the profiles of and modifiable dialysis practices associated with changes in DRT relative to the start of dialysis are unknown. We hypothesized hemodialysis (HD) dose and rates of intradialytic hypotension (IDH) would associate with changes in DRT in the first years after initiating dialysis.

**Methods:**

We analyzed data from adult HD patients who responded to a DRT survey ≤180 days from first date of dialysis (FDD) during 2014 to 2017. DRT survey was administered with annual KDQOL survey. DRT survey asks: “How long does it take you to be able to return to your normal activities after your dialysis treatment?” Answers are: < 0.5, 0.5-to-1, 1-to-2, 2-to-4, or > 4 h. An adjusted logistic regression model computed odds ratio for a change to a longer DRT (increase above DRT > 2 h) in reference to a change to a shorter DRT (decrease below DRT < 2 h, or from DRT > 4 h). Changes in DRT were calculated from incident (≤180 days FDD) to first prevalent (> 365-to- ≤ 545 days FDD) and second prevalent (> 730-to- ≤ 910 days FDD) years.

**Results:**

Among 98,616 incident HD patients (age 62.6 ± 14.4 years, 57.8% male) who responded to DRT survey, a higher spKt/V in the incident period was associated with 13.5% (OR = 0.865; 95%CI 0.801-to-0.935) lower risk of a change to a longer DRT in the first-prevalent year. A higher number of HD treatments with IDH episodes per month in the incident period was associated with a 0.8% (OR = 1.008; 95%CI 1.001-to-1.015) and 1.6% (OR = 1.016; 95%CI 1.006-to-1.027) higher probability of a change to a longer DRT in the first- and second-prevalent years, respectively. Consistently, an increased in incidence of IDH episodes/months was associated to a change to a longer DRT over time.

**Conclusions:**

Incident patients who had higher spKt/V and less sessions with IDH episodes had a lower likelihood of changing to a longer DRT in first year of HD. Dose optimization strategies with cardiac stability in fluid removal should be tested.

**Supplementary Information:**

The online version contains supplementary material available at 10.1186/s12882-020-02187-9.

## Background

Healthcare has begun to shift towards patient centered care models, which include adoption of various patient reported outcome (PROs) measures for routine assessment of individuals with end stage kidney disease (ESKD). Patients with ESKD can suffer a burden resulting not only from their kidney disease and comorbidities, but also from the dialysis treatment itself [1–3]. Capturing patients’ experiences related to routine dialysis care is an important step towards a more patient focused disease management [4].

Dialysis recovery time (DRT), defined as the perceived time to recover the ability to undertake normal activities after a dialysis, has been proposed as a meaningful variable to assess how the patient feels from and tolerates hemodialysis (HD) [5]. DRT has been shown to be a reliable marker of health-related quality of life (HRQOL) [5–15]. Prior studies indicate > 65% of prevalent HD patients report a DRT ≥2 h [9, 15]. Longer DRTs have been shown to be correlated to an array of symptoms including fatigue, and are predictors of hospitalization and mortality [6–9, 15]. Previous studies have shown DRT associates with a set of non-modifiable risk factors, such as age, serum albumin, diabetes and psychiatric disorders [15].

The pillars of the HD therapy are fluid removal and solute clearance, which we hypothesize could be modifiable factors that may lead to favorable changes to a shorter DRT [16] via episodes of intradialytic hypotension (IDH) and a reduction in uremic toxicity [17, 18]. Studies have reported frequent HD associates with changes to a shorter DRT as compared to trice weekly HD [5, 12]; this finding has been proposed to be driven by more hemodynamic stability and higher adequacy targets in frequent HD. However, the extent to which these findings are independent of achievements of higher dialysis adequacy and less IDH is unknown.

The profiles of DRT have not been defined in incident HD patients who recently progressed to ERSD and initiated renal replacement therapy (RRT). Furthermore, longitudinal trajectories of DRT have not been established, and it is unknown if practice patterns in trice weekly HD affect DRT. We aimed to longitudinally study the impacts of dialysis adequacy and IDH on changes in DRT, adjusted for multiple confounders, in a cohort of incident ESKD patients treated by HD. We hypothesized greater dialysis adequacy and a lower number of HD sessions with IDH episodes would be meaningfully associated with changes to a shorter DRT.

## Methods

### Study design

We conducted this study to investigate whether changes in DRT over the first 2 years of HD associate with dialysis adequacy and IDH. This research was conducted under a protocol reviewed by New England Independent Review Board, who confirmed the study was secondary research of existing patient data that were deidentified and did not require informed consent per title 45 of the United States Code of Federal Regulations part 46.104-d4ii (Needham Heights, MA; NEIRB#WO1–6614). The study was performed in adherence with the Declaration of Helsinki.

### Setting and participants

We used data previously collected from ESKD patients during standard-of-care HD at a large dialysis organization (LDO) in North America (Fresenius Kidney Care, Waltham, MA, United States) during January 2014 to December 2017. Analysis included data from adults (age ≥ 18 years at first date of dialysis (FDD)) treated via HD who completed ≥1 DRT survey within the first 180 days of RRT. We excluded patients age ≤ 18 years and pregnant females.

### DRT survey

DRT survey was a unique questionnaire used by the LDO. The DRT survey was administered conjunction with the Kidney Disease Quality of Life (KDQOL) survey. These questionnaires (KDQOL and additional DRT survey) were administered by the LDO as a standard of care in the incident dialysis period and annually thereafter.

The DRT survey includes one question and is completed by patients selecting one of five possible responses. The DRT survey question and answers are shown below.

“How long does it take you to be able to return to your normal activities after your dialysis treatment? (Circle the timeframe that best describes your answer)

1. Less than 30 min
2. Within 1 h
3. Within 1–2 h
4. Within 2–4 h
5. More than 4 h”

DRT survey was chosen by the LDO to be administered with the KDQOL to assess additional HRQOL parameters in June of 2013. By 2014 and through 2017, DRT survey was responded to by ≥99% of HD patients who completed the KDQOL survey.

### Primary outcome

The primary outcome of our study was the change in DRT from the incident period (≤180 days from FDD) to: 1) the first prevalent year (> 365-to- ≤ 545 days from FDD), and 2) second prevalent year (> 730-to- ≤ 910 days from FDD).

The exposure variables were the number of HD treatments with an IDH episode per month and dialysis adequacy (i.e. spKt/V). We considered the mean values for exposure variables ±30 days of the DRT survey date in the incident period and the change from the incident to the first/second year prevalent periods. A treatment with IDH episode was defined as ≥1 intradialytic systolic blood pressure (SBP) < 100 mmHg.

Covariates were selected in an a priori manner. These included the incident DRT, comorbidity burden (age, congestive heart failure (CHF), diabetes, ischemic heart disease (IHD), number of comorbidities and treatment schedule (majority of HD sessions started before/after 1200 h in the incident period and the change from incident to prevalent periods). For continuous variables, we used average values ±30 days of the DRT survey date. Categorical variables were determined from the most recent record to DRT survey date.

### Exploratory outcomes

We performed two descriptive exploratory analyses. First, we assessed if various demographic, environmental, comorbid, clinical, and laboratory parameters (detailed in supplemental files) were associated with DRT in the first 180 days of HD. Second, we investigated whether the DRT category in the first 180 days of HD was associated with crude 6-, 12-, and 24-month hospital admissions per patient per year (ppy).

### Statistical methods

Analyses were performed using SAS version 9.4 (SAS, Cary, NC, USA). Sankey diagrams and Forest plots constructed using R version 3.5.2 (R Foundation, Vienna, Austria).

#### Analysis of descriptive statistics

The characteristics of HD patients in the incident period was computed for demographic, comorbid, clinical, and laboratory parameters. Categorical and continuous variables were calculated as counts/proportions and mean ± standard deviation (SD). The changes from the incident DRT to the first- or second-prevalent year DRT were calculated as proportions and tabulated, as well as visualized via Sankey diagrams.

#### Analysis of primary outcome

Logistic regression models were constructed to calculate the odds ratio and confidence intervals for a change to a longer DRT from the incident to the first- and second- prevalent years.

We defined changes in DRT as a binary outcome with: 1) a change to a shorter DRT being any decrease from above to below a DRT category < 2 h, or a decrease from a DRT > 4 h to a lower DRT category (this was inclusive of patients who maintained a shorter DRT that was < 2 h), and 2) a change to a longer DRT being any increase from below to above a DRT category > 2 h (this was inclusive of patients who maintained a longer DRT with no reduction from the incident to follow up period). The selection of this binary outcome for changes in DRT was constructed considering a target time for DRT being < 2 h, but also considering any decrease in DRT in longer categories to be potentially favorable for the patients’ quality of life.

The exposure variables for the logistic regression models were dialysis adequacy, as measured by the spKt/V, and the number of HD treatments with IDH episodes per month; exposure variables included mean values in the incident period and the change in the mean values from the incident to the first/second year prevalent periods. We adjusted the models for incident DRT, age, CHF, diabetes, IHD and number of comorbidities.

#### Analysis of exploratory outcomes

For the first exploratory aim, we compared demographic, environmental, comorbid, clinical, and laboratory parameters by the DRT category in the first 180 days of HD. Comparisons were made in reference to a DRT < 0.5 h using two sample t-tests for means and Chi-Square methods for proportions. For the second exploratory aim, we constructed unadjusted Poisson models to compare hospital admission rates by DRT category in the first 180 days of HD; comparisons were made in reference to a DRT < 0.5 h.

## Results

### Patient characteristics

**Fig. 1:**
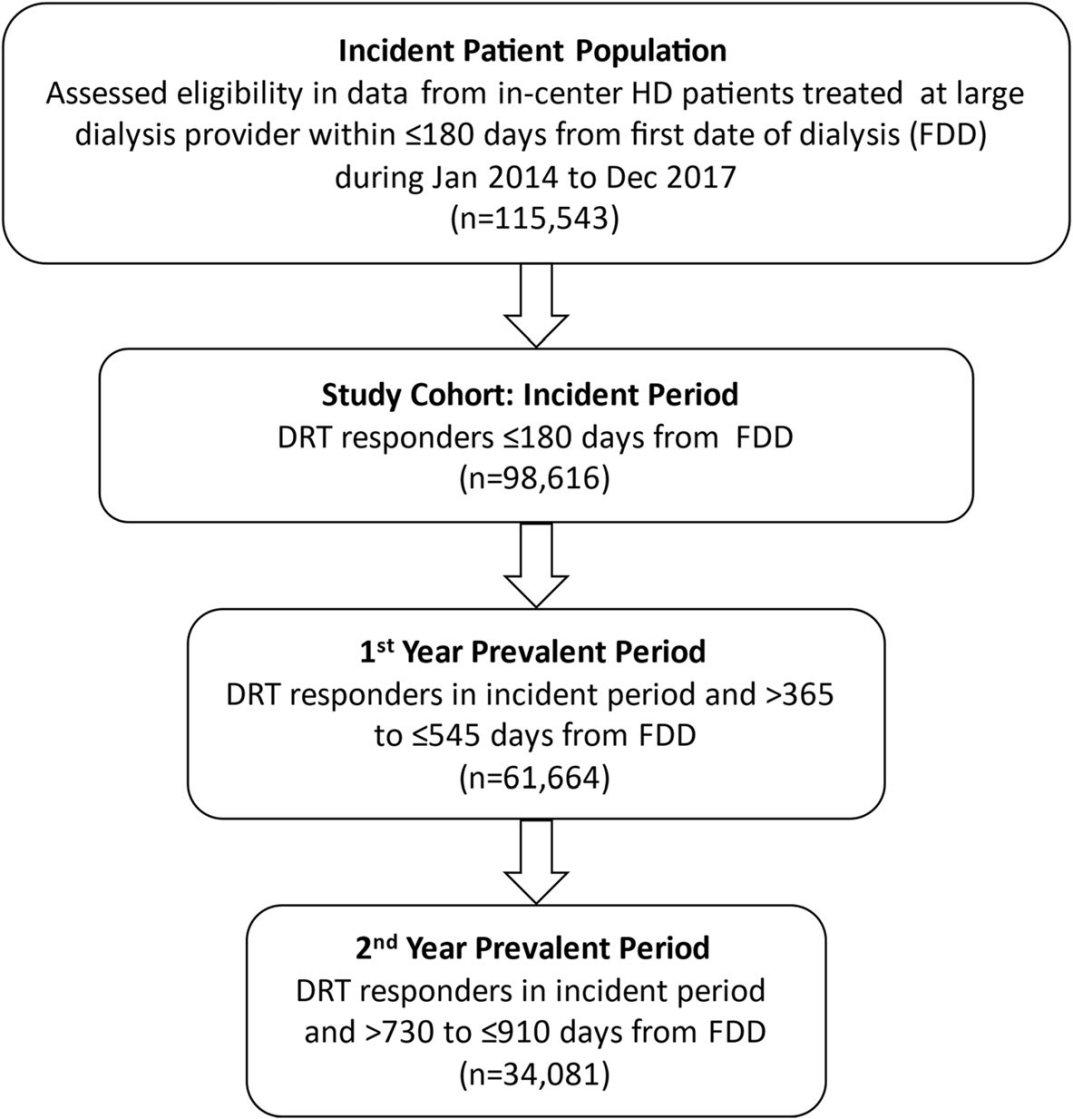
Patient Flow Diagram

We analyzed data from 98,616 patients who responded to DRT survey within 180 days from FDD (Fig. 1). This accounted for 85.4% of all incident HD patients in the study period. Patients were mean age 62.6 ± 14.4 years, 57.8% male, 69.1% white race, 64.7% used a dialysis catheter, 34.8% had diabetes, 14.8% had IHD, and 16.6% had CHF. Mean days to completion of the DRT survey was 112 ± 26 (Table 1).

**Table 1:** Characteristics of incident HD patients

Parameter	Overall Mean (SD) or N (%)
Demographics:	
Patient (n)	98,616 (100%)
Age (years)	62.6 (14.4)
Male (%)	56,991 (57.8%)
Race white (%)	58,528 (69.1%)
Non-Hispanic ethnicity (%)	72,293 (87.6%)
BMI (kg/m^2^)	29.4 (9.2)
Days from FDD to survey	112 (26)
Employed	11,643 (15.3%)
Comorbidities:	
Number of comorbidities (n)	1.92 (1.49)
Diabetes (%)	34,325 (34.8%)
Ischemic Heart Disease (%)	14,560 (14.8%)
Congestive Heart Failure (%)	16,377 (16.6%)
Treatment Parameters:	
HD start before 1200 h (%)	65,876 (66.8%)
UFV (L)	2.16 (0.92)
Normalized UFV by body weight (nUFVbw) (mL/kg)	26.6 (10.8)
UFR (mL/hour/kg)	7.06 (2.92)
HD treatment time (hours)	3.82 (0.44)
Number of treatments ±30 days of DRT survey (n)	24.55 (3.92)
Clinical Parameters:	
Catheter (%)	62,220 (64.7%)
Pre-HD weight (kg)	85.5 (23.7)
Post-HD weight (kg)	83.3 (23.3)
IDWG (kg)	2.21 (0.93)
Treatments with IDH episodes (SBP < 100) per month (n)	3.01 (3.32)
Laboratories:	
Pre-HD BUN (mg/dL)	53.9 (16.6)
Post-HD BUN (mg/dL)	14.1 (5.7)
Kt/V	1.624 (0.318)
Albumin (g/dL)	3.67 (0.42)
Sodium (mmol/L)	137.9 (3.1)
Potassium (mmoI/L)	4.54 (0.55)
Glucose (mg/dL)	183.5 (88.0)
Calcium (mg/dL)	8.95 (0.60)
Phosphate (mg/dL)	5.21 (1.32)
iPTH (pg/mL)	386.5 (287.7)
Hemoglobin (g/dL)	10.83 (0.99)
White blood cells (10^9^/L)	7.18 (2.51)
Neutrophil to lymphocyte ratio	4.21 (3.16)

*HD* hemodialysis, *DRT* dialysis recovery time, *BMI* Body mass index, *FDD* First date of dialysis, *UFV* Ultrafiltration volume, *UFR* Ultrafiltration rate, *IDWG* Intradialytic weight gain, *IDH* intradialytic hypotension, *BUN* Blood urea nitrogen, *iPTH* Intact parathyroid hormone

### Profiles of DRT in incident patients

Incident patients in the first 180 days of HD typically had a DRT < 1 h or > 4 h. Rates of DRT in incident patients were: 25.2% DRT < 0.5 h, 19.1% DRT 0.5-to-1 h, 17.3% 1-to-2 h, 15.5%, 2-to-4 h, and 22.9% DRT > 4 h.

In the exploratory analysis of patient characteristics by DRT category in the first 180 days of HD, we observed most demographic, environmental, comorbid, clinical, and laboratory parameters were distinct between DRT categories, as compared to a DRT < 0.5 h (Additional file 1: Supplemental Table 1).

In the exploratory assessment of hospital admission rates associated with DRT categories, we observed unadjusted 6-, 12-, and 24-month hospital admission rates ppy rose with each longer DRT category (all *p* < 0.001), as compared to rates in patients with a DRT < 0.5 h (Additional file 2: Supplemental Fig. 1).

### Trajectories of DRT

**Fig. 2:**
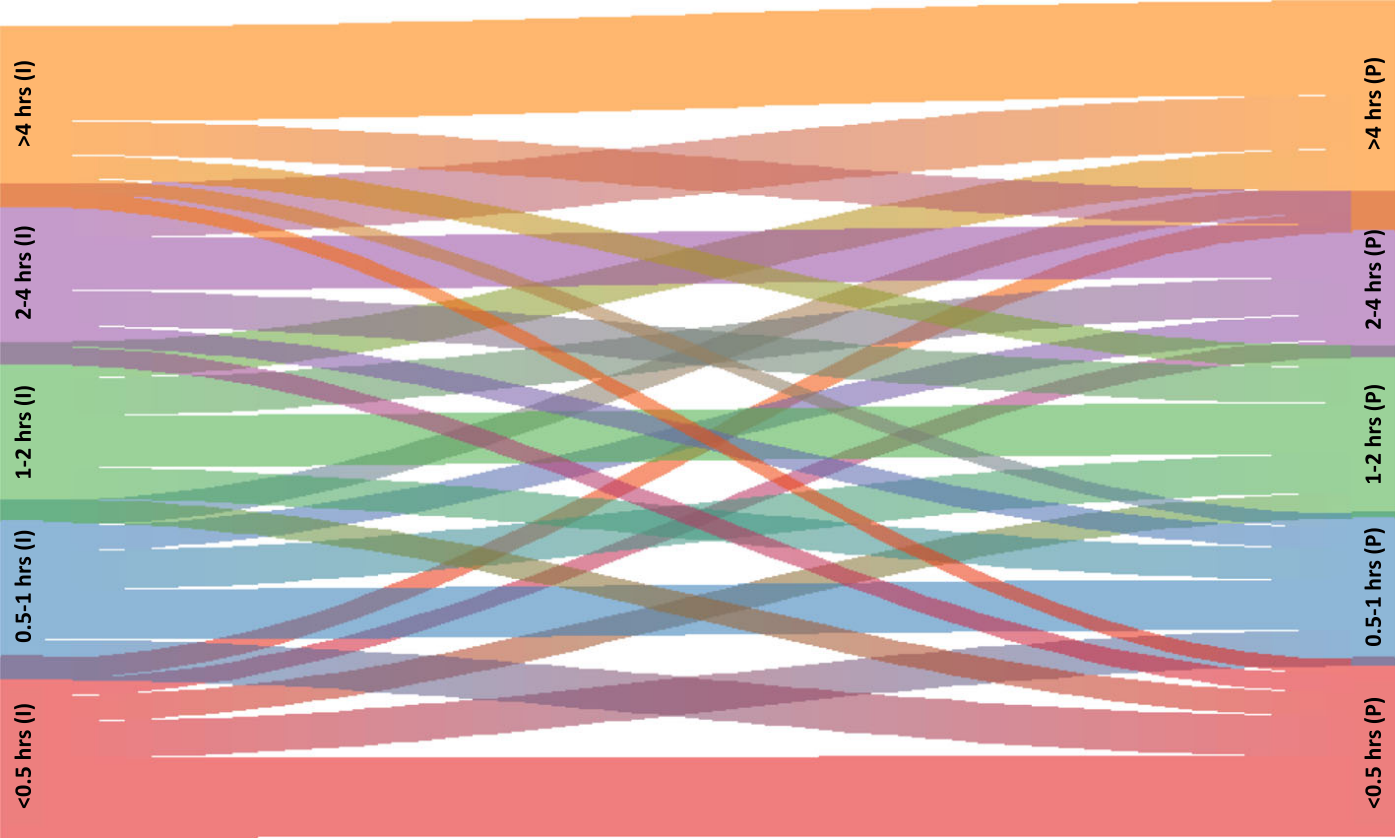
Sankey diagram river plot of the changes in DRT from ≤180 days FDD to > 365-to- ≤ 545 days FDD. (I) = incident DRT from ≤180 days FDD on left; (P) = first year prevalent DRT from > 365-to- ≤ 545 days FDD on right

Changes in DRT categories from the incident period (≤180 days from FDD) to subsequent periods in the first prevalent year (> 365-to- ≤ 545 days from FDD) or the second prevalent year (> 730-to- ≤ 910 days from FDD) are presented in Table 2 and visualized with Sankey diagram river plots in Fig. 2 and Additional file 3: Supplemental Fig. 2. About 40% of patients with an incident DRT < 0.5 h, and about 50% of patients with incident DRT > 4 h, remained in the same DRT category in the first- and second-year prevalent periods. In the Sankey diagram, this is shown via the thickest rivers/flows that remained in the same DRT category. DRT typically changed into categories < 0.5 h or > 4 h, with the largest proportion of patients that changed into the > 4 h category; this is shown in the Sankey diagram by thicker rivers that increased, thinner rivers that decreased, and the thickest river sections at the right of the graphics.

**Table 2:**
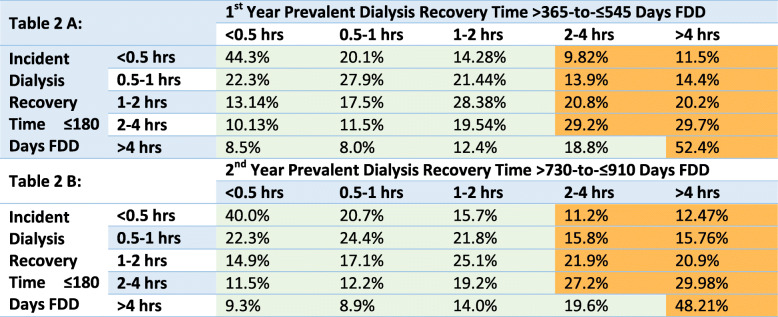
Changes in dialysis recovery time in the first years on HD

Shaded cells denote method used for categorizing DRT changes for the logistic regression models. Green shaded cells denote patient group with a change to a shorter DRT; this group was inclusive of patients who maintained a shorter DRT that was < 2 h. Orange shaded cells denote patient group with changes to a longer DRT; this group was inclusive of patients who maintained a longer DRT with no reduction from the incident to follow up period

### Impacts of dialysis adequacy and IDH on changes in DRT

For the logistic regression models assessing the impacts of dialysis adequacy and IDH on changes in DRT, we categorized changes in DRT in a binary manner as: 1) a change to a shorter DRT (green shaded cells; Table 2), or 2) a change to a longer DRT (orange shaded cells; Table 2). The model was designed to determine the probability of covariates being associated with changing to an longer DRT in reference to a shorter DRT.

**Fig. 3:**
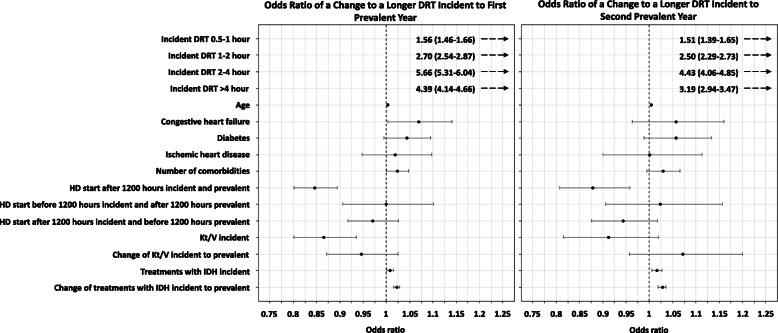
Forest plots of odds ratio and confidence intervals for variables associated to a change to a longer DRT in reference to a change to a shorter DRT

The adjusted logistic regression models to test our hypothesis revealed a higher dialysis adequacy in the incident period was associated with a 13.5% (OR = 0.865;95%CI 0.801-to-0.935) lower odds of an longer DRT in the first prevalent year of HD (Fig. 3; Tables 3 and 4). A higher number of HD treatments with IDH episodes per month in the incident period was associated with a 0.8% (OR = 1.008;95%CI 1.001-to-1.015) and 1.6% (OR = 1.016;95%CI 1.006-to-1.027) higher probability of an longer DRT in the first- and second-prevalent years, respectively. Consistently, a change to a higher number of treatments with IDH episodes/month in the incident to prevalent periods was associated with a 2.2% (OR = 1.022;95%CI 1.015-to-1.028) and 2.8% (OR = 1.028;95%CI 1.019-to-1.036) higher likelihood of an longer DRT in the first- and second-prevalent years, respectively.

**Table 3:** Odds ratio of a change to a longer DRT from the incident to first prevalent year

Parameter	Odds Ratio	95% Confidence Limits
Incident DRT 0.5–1 h	1.557	1.463 to 1.657
Incident DRT 1–2 h	2.698	2.536 to 2.871
Incident DRT 2–4 h	5.661	5.309 to 6.037
Incident DRT > 4 h	4.388	4.137 to 4.655
Age	1.003	1.002 to 1.004
Congestive heart failure	1.07	1.003 to 1.141
Diabetes	1.044	0.995 to 1.095
Ischemic heart disease	1.019	0.947 to 1.097
Number of comorbidities	1.024	1.001 to 1.048
HD start after 1200 h incident and prevalent	0.846	0.801 to 0.894
HD start before 1200 h incident and after 1200 h prevalent	0.999	0.906 to 1.101
HD start after 1200 h incident and before 1200 h prevalent	0.97	0.917 to 1.026
Kt/V incident	0.865	0.801 to 0.935
Δ Kt/V incident to prevalent	0.946	0.872 to 1.025
Treatments with IDH episodes per month incident	1.008	1.001 to 1.015
Δ Treatments with IDH episodes per month incident to prevalent	1.022	1.015 to 1.028

**Table 4:** Odds ratio of a change to a longer DRT incident to second prevalent year

Parameter	Odds Ratio	95% Confidence Limits
Incident DRT 0.5–1 h	1.512	1.388 to 1.646
Incident DRT 1–2 h	2.500	2.294 to 2.725
Incident DRT 2–4 h	4.434	4.057 to 4.846
Incident DRT > 4 h	3.192	2.939 to 3.467
Age	1.004	1.002 to 1.006
Congestive heart failure	1.057	0.963 to 1.160
Diabetes	1.058	0.988 to 1.133
Ischemic heart disease	1.001	0.900 to 1.113
Number of comorbidities	1.03	0.995 to 1.066
HD start after 1200 h incident and prevalent	0.879	0.807 to 0.959
HD start before 1200 h incident and after 1200 h prevalent	1.024	0.907 to 1.156
HD start after 1200 h incident and before 1200 h prevalent	0.944	0.876 to 1.017
Kt/V incident	0.912	0.816 to 1.020
Δ Kt/V incident to prevalent	1.072	0.957 to 1.200
Treatments with IDH episodes per month incident	1.016	1.006 to 1.027
Δ Treatments with IDH episodes per month incident to prevalent	1.028	1.019 to 1.036

The covariates included in the adjusted analysis further identified the predominant determinant of a longer DRT in both the prevalent periods was the incident DRT value. Patients with an incident DRT category of > 0.5 h had > 50%, and up to a fivefold higher probability of a change to a longer DRT category in both prevalent periods (all *p* < 0.001). A longer DRT was also associated with an older age in both years (both *p* < 0.01), CHF in the first-prevalent year (*p* < 0.05), and a higher number of comorbidities in the first-prevalent year (*p* ≤ 0.05). Diabetes and IHD were not associated to changes in DRT.

Patients with most of their HD start times after noon (1200 h) at the incident and prevalent periods had a 15% (OR = 0.846; 95%CI 0.801-to-0.894) and a 12% (OR = 0.879;95%CI 0.807-to-0.959) lower likelihood of an longer DRT in both prevalent years. However, changing from a HD start time before 1200 h in the incident period to after 1200 h in the first- and second-prevalent periods, and vice versa, was not associated to changes in DRT.

## Discussion

In this retrospective study, we found DRT was generally reported to be < 1 h or > 4 h in the first 180 days after initiation of RRT. Given the DRT survey was completed by 85% of all patients at the LDO within the first 180 of HD, these findings are representative the provider’s national population. We described unique longitudinal trajectories of DRT in patients who survived the follow-up periods. Most HD patients progressed to longer DRT categories during the first 2 years of HD. This unfortunately suggests patients may tend to feel progressively worse from their lifesaving HD treatments over time. For our hypothesis-derived endpoint, we found a higher dialysis adequacy and a lower number of IDH episodes per month in the incident period was associated with patients having a change to a shorter DRT, or a sustained shorter DRT that was < 2 h. It appears that it will be important for clinicians and providers to strive to achieve a high removal of retention solutes without causing IDH to optimize how patients feel from their HD treatments.

To account for the cumulative nature of cardiovascular and inflammatory dysfunctions over time in ESKD in this study, we postulated the hypothetical drivers of longer DRT and constructed longitudinal models that considered modifiable factors which may have the potential to improve DRT [2, 16, 19]. In that sense, this study focused on solute clearance and IDH as important modifiable factors. Ultimately, we found achievement of higher spKt/V and less episodes of IDH provided a higher probability of a change to a shorter DRT from the incident to 1-year prevalent HD period. A study of DRT over time in 364 HD patients (mean vintage 2.4 years) suggested the factors associated with a higher DRT over time included HD vintage > 6 months, high BMI, low post-HD SBP, and intradialytic cramping, post-SBP < 115 mmHg, albeit the study did not disclose any precise window of exposure [10]. Contrary to our findings, a cross sectional analysis of the DOPPS data did not identify an association between Kt/V and DRT [15]. The findings in the DOPPS data might be representative of the prevalent cohort studied (mean dialysis vintage of 3.3 years), and this is consistent with our results in the second prevalent year of HD.

Modalities such as frequent HD and hemodiafiltration (HDF) can achieve a high adequacy and more fluid removal while maintaining hemodynamic stability, which may lead to better DRT [5, 12–14]. The Frequent Hemodialysis Network (FHN) trial and observational studies have demonstrated frequent HD leads to lower DRT compared to thrice weekly HD [5, 12]. The effect sizes for reductions in DRT from frequent HD in the FHN trial were up to 60 min, which translates into meaningful improvements for patients [12]. A randomized cross-over trial found HDF does not promote better DRT compared to HD [14]. Interestingly, the Kt/V and ultrafiltration volume was similar between the HDF and HD groups and the symptomatic IDH rate was higher in HDF versus HD, which might have influenced the trial’s overall negative findings [14]. In the context of the results of our study, it appears achievement of a high spKt/V with hemodynamic stability throughout the first year of RRT may be of high importance to how the patient feels after HD. However, a high Kt/V may not be as meaningfully associated with DRT among patients who survive several years on dialysis.

In our exploratory analysis of the associations between patient characteristics and DRT in the first 180 days of HD, we found longer DRT categories were associated with several markers of comorbidity burden and poor fluid control, which is consistent with prior studies in the prevalent HD patients [11, 15, 20]. BMI and female sex were associated with longer DRTs reported in incident HD patients. Interestingly, we also observed that patients who start HD before 1200 h reported longer incident DRT. Although HD schedule may influence DRT, findings could be confounded, and patients being treated in the evening/night shifts may represent a subgroup with better functional status who perceive shorter DRT. Sleep patterns may also contribute to the findings, given late shift patients may sleep soon after HD and perceive a shorter DRT, albeit this relationship remains underexplored [16].

Although this study has many strengths including use of large population that is representative of HD patients treated in the United States, there are some key limitations. The DRT survey used by the provider was unique and has not been formally validated against other DRT survey options [5, 12, 15]. Nonetheless, in an exploratory analysis we found patients who reported a longer DRT category in the incident period had higher unadjusted 6-, 12- and 24-month hospitalization rates, which is similar to prior findings in prevalent HD [15]. Other limitations of our study include not accounting for dialysate sodium, which has been found to be inversely associated to DRT presumably on account of higher hemodynamic instability [15, 21, 22]. However, the gradient in blood/dialysate sodium and potential variations in intradialytic tonicity might be independently associated to longer DRT. Also, higher sodium dialysate may cause more residual fluid overload, which could longitudinally associate with longer DRT. Residual kidney function was not taken into account, which may lead to better fluid control, solute clearance and a shorter DRT [10, 23]. Moreover, our model may not have optimized ordinal information provided by DRT categorization, which could restrict our power to detect smaller, but still clinically meaningful changes. Additionally, in this longitudinal study design, we cannot rule out selection bias conditioned by survival. Also, we cannot rule out residual confounding due to observational nature of the study. Particularly, our models did not include covariates such as cognitive function, psychiatric comorbidities and employment status, which could be potential confounders. Finally, although we had a relatively large sample size, our results may not be generalizable to different countries or other heterogeneous populations.

Despite some limitations, this evaluation of novel modifiable determinants of longitudinal changes in DRT has many strengths. Variables were selected in an a priori model in which dialysis adequacy and IDH were hypothesized to be consistent and clinically meaningful variables influencing DRT, thereby mapping potential interventions [24]. The longitudinal design minimizes systematic errors, particularly reverse causality in association between IDH and DRT. We adjusted for key confounders and described longitudinal associations that warrant interventional testing.

## Conclusion

In conclusion, this novel description of trajectories of changes in DRT in incident HD patients suggest targeting higher spKt/V while limiting IDH may have the potential to lead to a shorter DRT from the incident to prevalent years. The majority of incident HD patients tend to report longer DRT after 2 years. Our findings support the concept that higher solute removal and optimal fluid control may improve DRT in HD patients, which may be tested in appropriately designed clinical trials in the future.

## Supplementary Information

**Additional file 1: Supplemental Table 1.** Exploratory analysis of the determinants of DRT in incident HD patients.

**Additional file 2: Supplemental Fig.ure 1.** Exploratory analysis of unadjusted 6-, 12-, and 24-month hospital admission rates by DRT category in incident HD patients.

**Additional file 3: Supplemental Figure 2.** Sankey diagram river plot of the changes in DRT from ≤180 days FDD to > 730-to- ≤ 910 days FDD. (I) = incident DRT from ≤180 days FDD on left; (P) = second year prevalent DRT from > 730-to- ≤ 910 days FDD on right.

**Additional file 4 :** STROBE Checklist.

## Data Availability

The datasets and coding utilized for this study are not publicly available. The datasets were obtained from the Fresenius Medical Care North America Knowledge Center Data Warehouse, which is restricted to use by only authorized employees and is not publicly available.

## Abbreviations

DRT: Dialysis Recovery Time
HD: Hemodialysis
IDH: Intra-dialytic hypotension
FDD: First day of dialysis
HDF: Hemodiafiltration
FHN: Frequent Hemodialysis Network
UFV: Ultra filtration volumes
UFR: Ultra filtration rates
HRQOL: Health-related quality of life
RRT: Renal replacement therapy
