# Supplementary material for: Impacts of dialysis adequacy and intradialytic hypotension on changes in dialysis recovery time
Source: BMC Nephrol. 2020 Dec 7;21:529. doi: 10.1186/s12882-020-02187-9 (PMC7720452; doi:10.1186/s12882-020-02187-9)
Supplement: Supplementary file 1 — Additional file 1: Supplemental Table 1. Exploratory analysis of the determinants of DRT in incident HD patients. [file 12882_2020_2187_MOESM1_ESM.docx]

**Additional File 1: Supplemental Table 1:**

| Supplemental Table 1: Determinants of Dialysis Recovery Time in Incident HD Patients | | | | | | |
| --- | --- | --- | --- | --- | --- | --- |
| Parameter | **Overall**  Mean (SD) or N (%) | **Dialysis Recovery Time ≤180 Days FDD (hours)**  Mean (SD), Column %, or Rate PPY | | | | |
| Demographics: | | **<0.5 (Reference)** | **0.5-1** | **1-2** | **2-4** | **>4** |
| Patient (n) | 98616 (100%) | 25.2% | 19.1% | 17.3% | 15.5% | 22.9% |
| Age (years) | 62.6 (14.4) | 62.9 (14.5) | 62.7 (14.5) | **62.4*** (14.5)** | **62.1*** (14.4)** | 62.8 (13.9) |
| Male (%) | 56991 (57.8%) | 62.6% | **57.3%***** | **56.7%***** | **55.5%***** | **55.3%***** |
| Race white (%) | 58528 (69.1%) | 66.5% | 66.4% | **68.3%***** | **69.1%***** | **74.6%***** |
| Non-Hispanic ethnicity (%) | 72293 (87.6%) | 86.7% | 86.2% | 87.2% | **88.5%***** | **89.6%***** |
| BMI (kg/m^2^) | 29.4 (9.2) | 28.9 (8.9) | 29.1 (9.2) | **29.3*** (9.2)** | **29.7*** (9.3)** | **30.0*** (9.6)** |
| Days from FDD to survey | 112 (26) | 110 (26) | 112 (26) | 112 (26) | **113* (26)** | **113** (26)** |
| Employed | 11643 (15.3%) | 18.2% | **15.7%***** | **14.7%***** | **13.9%***** | **13.0%***** |
| Environmental Parameters: | | | | | | |
| Resides in same zip code as clinic (%) | 25707 (26.1%) | 25.7% | **26.8%*** | **26.6%*** | 26.4% | 25.4% |
| Season: spring (%) | 25957 (26.3%) | 26.0% | **27.0%^a^** | 26.4% | 26.4% | 26.0% |
| Season: summer (%) (*Reference*) | 26425 (26.8%) | 26.7% | 26.2% | 27.0% | 26.8% | 27.2% |
| Season: fall (%) | 24397 (24.7%) | 24.6% | 24.5% | 24.9% | 24.4% | 25.2% |
| Season: winter (%) | 21837 (22.1%) | 22.7% | 22.3% | **21.7%^a^** | 22.4% | **21.6%^aa^** |
| Differences in columns between all 4 seasons | *P-Value* | *Reference* | 0.131 | 0.086 | 0.808 | **0.014*** |
| Comorbidities: | | | | | | |
| Number of comorbidities (n) | 1.92 (1.49) | 1.88 (1.47) | 1.89 (1.47) | **1.91* (1.48)** | **1.92** (1.49)** | **2.00*** (1.55)** |
| Diabetes (%) | 34325 (34.8%) | 33.6% | 34.3% | **35.2%***** | **35.0%**** | **36.1%***** |
| Ischemic Heart Disease (%) | 14560 (14.8%) | 14.7% | 14.1% | 14.5% | 14.3% | **16.0%***** |
| Congestive Heart Failure (%) | 16377 (16.6%) | 16.0% | 16.5% | 16.5% | 16.2% | **17.7%***** |
| Treatment Parameters: | | | | | | |
| HD start before 1200 hours (%) | 65876 (66.8%) | 63.1% | **65.3%***** | **69.2%***** | **72.3%***** | **66.6%***** |
| UFV (L) | 2.16 (0.92) | 2.17 (0.92) | 2.17 (0.90) | **2.15* (0.91)** | 2.16 (0.91) | **2.14*** (0.93)** |
| Normalized UFV by body weight (nUFVbw) (mL/kg) | 26.6 (10.8) | 26.9 (10.8) | 27.1 (10.9) | **26.6 (10.8)*** | **26.5 (10.9)***** | **25.9 (10.8)***** |
| UFR (mL/hour/kg) | 7.06 (2.92) | 7.14 (2.90) | **7.19* (2.93)** | **7.07* (2.92)** | **7.03*** (2.93)** | **6.87*** (2.91)** |
| HD treatment time (hours) | 3.82 (0.44) | 3.82 (0.43) | 3.82 (0.42) | 3.82 (0.42) | 3.83 (0.45) | 3.83 (0.45) |
| Number of treatments ±30 days of DRT survey (n) | 24.55 (3.92) | 24.71 (3.85) | **24.61* (3.84)** | **24.62* (3.65)** | **24.61**(3.94)** | **24.23*** (4.20)** |
| Clinical Parameters: | | | | | | |
| Catheter (%) | 62220 (64.7%) | 63.4% | 64.2% | **65.3%***** | **64.8%**** | **65.8%***** |
| Pre-HD weight (kg) | 85.5 (23.7) | 84.8 (22.9) | **84.2* (23.2)** | 85.1 (23.8) | **86.2*** (23.9)** | **87.1*** (24.7)** |
| Post-HD weight (kg) | 83.3 (23.3) | 82.6 (22.5) | **82.1* (22.8)** | 83.0 (23.3) | **84.0*** (23.4)** | **85.0*** (24.2)** |
| IDWG (kg) | 2.21 (0.93) | 2.22 (0.94) | 2.22 (0.92) | 2.20 (0.92) | 2.22 (0.92) | 2.20 (0.95) |
| Treatments with IDH episodes (SBP<100) per month (n) | 3.01 (3.32) | 2.87 (3.28) | **2.96** (3.29)** | **3.04*** (3.29)** | **3.09*** (3.37)** | **3.15*** (3.39)** |
| Laboratories: | | | | | | |
| Pre-HD BUN (mg/dL) | 53.9 (16.6) | 54.5 (16.5) | **54.2* (16.6)** | **53.6*** (16.6)** | **53.6*** (16.4)** | **53.3*** (16.7)** |
| Post-HD BUN (mg/dL) | 14.1 (5.7) | 14.4 (5.8) | **14.1*** (5.8)** | **13.9*** (5.7)** | **13.9*** (5.5)** | **13.9*** (5.7)** |
| Kt/V | 1.624 (0.318) | 1.609 (0.313) | **1.630*** (0.319)** | **1.632*** (0.321)** | **1.627*****  **(0.315)** | **1.625*** (0.322)** |
| Albumin (g/dL) | 3.67 (0.42) | 3.71 (0.41) | **3.68*** (0.41)** | **3.67*** (0.42)** | **3.66*** (0.42)** | **3.64*** (0.43)** |
| Sodium (mmol/L) | 137.9 (3.1) | 138.0 (3.1) | **137.9*** (3.1)** | **137.8*** (3.1)** | **137.8*** (3.1)** | **137.7*** (3.2)** |
| Potassium (mmoI/L) | 4.54 (0.55) | 4.56 (0.55) | 4.55 (0.55) | **4.54*** (0.55)** | **4.53*** (0.54)** | **4.54* (0.55)** |
| Glucose (mg/dL) | 183.5 (88.0) | 177.3 (84.1) | **181.3** (86.2)** | **185.0*** (88.6)** | **184.9*** (87.7)** | **189.8*** (92.7)** |
| Calcium (mg/dL) | 8.95 (0.60) | 8.93 (0.59) | **8.95* (0.60)** | **8.95* (0.59)** | **8.96*** (0.60)** | **8.95** (0.60)** |
| Phosphate (mg/dL) | 5.21 (1.32) | 5.21 (1.33) | 5.20 (1.32) | 5.19 (1.32) | 5.24 (1.31) | 5.23 (1.33) |
| iPTH (pg/mL) | 386.5 (287.7) | 396.1 (291.1) | 390.7 (290.0) | **389.5* (292.9)** | **383.7*** (283.7)** | **372.1*** (280.0)** |
| Hemoglobin (g/dL) | 10.83 (0.99) | 10.87 (0.98) | **10.82*** (0.99)** | **10.83*** (1.00)** | **10.82*** (0.98)** | **10.80*** (1.01)** |
| White blood cells (10^9^/L) | 7.18 (2.51) | 6.98 (2.38) | **7.11*** (2.60)** | **7.20*** (2.62)** | **7.25*** (2.38)** | **7.41*** (2.57)** |
| Neutrophil to lymphocyte ratio | 4.21 (3.16) | 4.12 (3.02) | 4.17 (3.06) | 4.17 (3.07) | **4.24** (3.30)** | **4.37*** (3.36)** |
| Differences in means/proportions/rates with DRT of <0.5 hours as the reference. *, p<0.05; **, p<0.01; ***, p<0.001; Differences between seasons were made with summer as the reference and are in reference to a DRT of <0.5 hours. ^a^, p<0.05; ^aa^, p<0.01  HD: hemodialysis; DRT: dialysis recovery time; BMI: Body mass index; FDD: First date of dialysis; UFV: Ultrafiltration volume; UFR; Ultrafiltration rate; IDWG: Intradialytic weight gain; IDH: intradialytic hypotension; BUN: Blood urea nitrogen; iPTH: Intact parathyroid hormone.  Continuous variables used average values ±30 days of the DRT survey date. Categorical variables used the most recent record to DRT survey date. UFV was calculated [Δ pre-HD – post-HD weight]. nUFVbw was calculated [((Δ pre-HD – post-HD weight)*1000)/post-HD weight]. UFR was calculated [(((Δ pre-HD – post-HD weight)*1000)/(HD time/60))/post-HD weight]. | | | | | | |
