# Supplementary material for: Impacts of dialysis adequacy and intradialytic hypotension on changes in dialysis recovery time
Source: BMC Nephrol. 2020 Dec 7;21:529. doi: 10.1186/s12882-020-02187-9 (PMC7720452; doi:10.1186/s12882-020-02187-9)
Supplement: Supplementary file 2 — Additional file 2: Supplemental Fig.ure 1. Exploratory analysis of unadjusted 6-, 12-, and 24-month hospital admission rates by DRT category in incident HD patients. [file 12882_2020_2187_MOESM2_ESM.docx]

**Additional File 2: Supplemental Figure 1:**


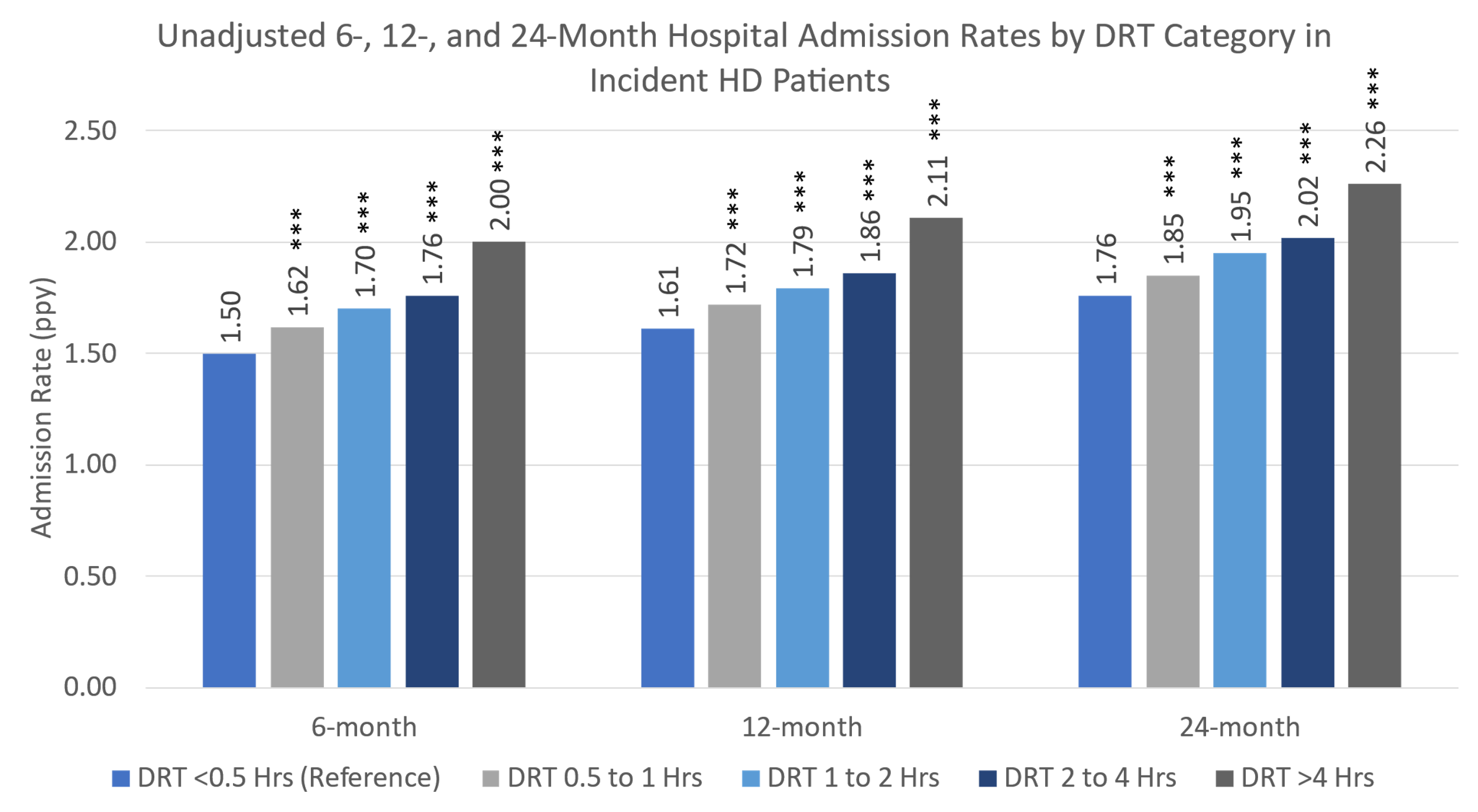


**Supplemental Figure 1:** Exploratory analysis of unadjusted 6-, 12-, and 24-month hospital admission rates by DRT category in incident HD patients. ***, p<0.001.
