# Supplementary material for: Impacts of dialysis adequacy and intradialytic hypotension on changes in dialysis recovery time
Source: BMC Nephrol. 2020 Dec 7;21:529. doi: 10.1186/s12882-020-02187-9 (PMC7720452; doi:10.1186/s12882-020-02187-9)
Supplement: Supplementary file 3 — Additional file 3: Supplemental Figure 2. Sankey diagram river plot of the changes in DRT from ≤180 days FDD to > 730-to- ≤ 910 days FDD. (I) = incident DRT from ≤180 days FDD on left; (P) = second year prevalent DRT from > 730-to- ≤ 910 days FDD on right. [file 12882_2020_2187_MOESM3_ESM.docx]

**Additional File 3: Supplemental Figure 2:**


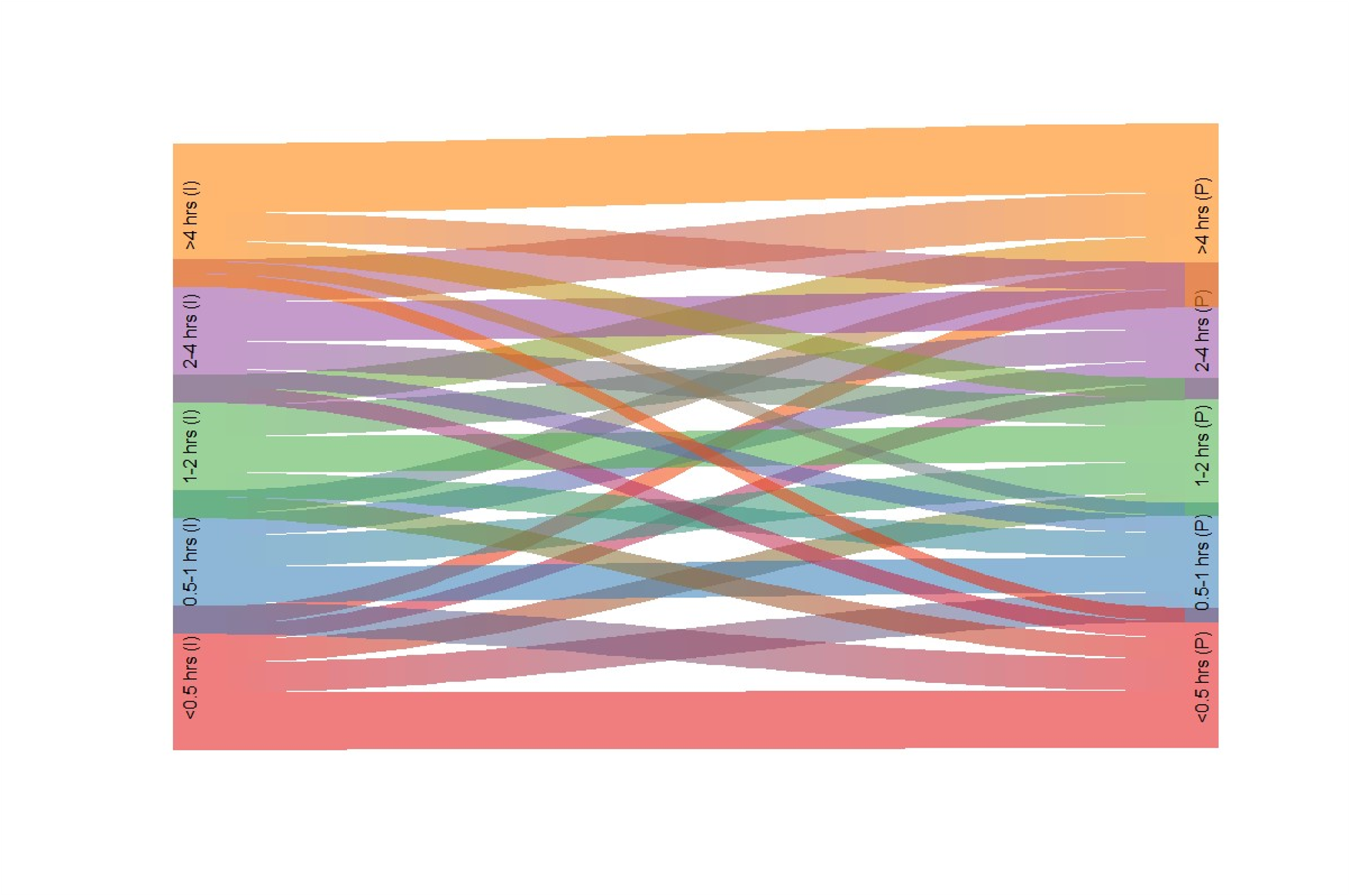


**Supplemental Figure 2:** Sankey diagram river plot of the changes in DRT from ≤180 days FDD to >730-to-≤910 days FDD. (I)= incident DRT from ≤180 days FDD on left; (P)=second year prevalent DRT from >730-to-≤910 days FDD on right.
